# Blood pressure screening in Mata Sector, a rural area of Rwanda

**DOI:** 10.1038/s41371-024-00912-7

**Published:** 2024-04-24

**Authors:** Isabella Hunjan, Alice Umulisa, Gianfranco Parati, Mario G. Bianchetti, Gregorio P. Milani, Bienvenu Muvunyi, Evariste Ntaganda, Dragana Radovanovic, Clara Stroppa, Paolo Suter, Franco Muggli

**Affiliations:** 1 School of Medicine and Surgery, University of Milano-Bicocca, Milan, Italy; 2 Family Medicine Institute, Faculty of Biomedical Sciences, Università della Svizzera Italiana, Lugano, Switzerland; 3 Health Care Centre of Nyamyumba, District of Nyaruguru, Nyamyumba, Rwanda; 4 Department of Cardiovascular, Neural and Metabolic Sciences, Istituto Auxologico Italiano IRCCS, San Luca Hospital, Milan, Italy; 5 Pediatric Unit, Fondazione IRCCS Ca’ Granda Ospedale Maggiore Policlinico, Milan, Italy; 6 Department of Clinical Sciences and Community Health, Università degli Studi di Milano, Milan, Italy; 7 Medical Specialized Services, King Faisal Hospital, Kigali, Rwanda; 8 Cardiovascular diseases Unit, Non-communicable diseases Division, Rwanda Biomedical Center, Kigali, Rwanda; 9 Department of Internal Medicine, University Hospital of Zurich, Zurich, Switzerland

**Keywords:** Hypertension, Lifestyle modification

## Abstract

In rural sub-Saharan Africa, knowledge of non-communicable diseases such as high blood pressure (BP) is rather limited. This report provides information about a BP screening in Mata Sector, a rural region in Southern Province of Rwanda. Community-based, house-to-house screening was performed between February and July 2020 on more than 7000 inhabitants. The screening was conducted by a local team composed by 20 community health care workers, five community health care supervisors, and one nurse with hypertension surveillance training. BP and heart rate were recorded after 5 min of resting, using a validated automated oscillometric OMRON M6 IT-HEM-7322-E monitor with Intelli Wrap Cuff (HEM-FL31-E) technology. The mean of the second and third value was retained. BP was normal (<140/90 mm Hg) in 6340 (88%) and elevated in 863 (12%) participants with 95% of unawareness. Grade 1 (140–159/90–99 mm Hg) hypertensive BP readings were detected in 697 (81%), grade 2 (160–179/100–109 mm Hg) in 134 (16%), and grade 3 (≥180/≥110 mm Hg) in 32 (3.7%) individuals. The prevalence of hypertensive readings was significantly age-dependent. Additionally, a slightly greater proportion of participants with high BP (14% versus 11%) had a body mass index (BMI) ≥ 25.0 kg/m^2^. Also resting heart rate was higher in individuals with high BP (82 versus 77 beats/min). Although individuals identified with occasionally elevated BP values need further confirmatory measurements to establish the diagnosis of hypertension, these data suggest that high BP represents a noteworthy and preventable reason of concern within sub-Saharan Africa.

## Introduction

In sub-Saharan Africa, infectious diseases like malaria, tuberculosis, and AIDS have historically been major causes of illness. However, countries within this region, as many low- and middle-income ones worldwide, are experiencing a shift in disease patterns. Communicable and childhood-related diseases are nowadays overshadowed by chronic, non-communicable diseases [1, 2]. Cancer, chronic respiratory diseases, diabetes, mental disorders, and cardiovascular diseases including rheumatic heart disease and arterial hypertension, are becoming increasingly burdensome in several countries in sub-Saharan Africa [3]. Knowledge of these non-communicable diseases in sub-Saharan Africa is rather limited, particularly in remote rural areas. This report provides information about a preliminary blood pressure (BP) screening performed in Mata Sector, a rural region in Southern Province of Rwanda.

## Methods

### General Information

Mata Sector is located in Nyaruguru District, Southern Rwanda, approximately 27.5 km west of Huye, the main urban center of this Province. It is a rural region with a population of around 15,000 people (2020: 15,691) spread across an area of 62.2 km^2^ at an elevation of approximately 1900 meters above sea level. It has a subtropical highland climate, characterized by steadily moderate temperatures and rainfall evenly spread throughout the year. Within Mata, there are 16 villages that prioritize sustainable farming methods and community development projects. The Association Mabawa - Wings for Africa (https://en.mabawa.org/chisiamo) in Mata Sector since 2005 has had a profound and transformative role in this region, which was deeply impacted by the 1994 Genocide against the Tutsi [4]. This association has promoted numerous initiatives that have significantly improved the quality of life in the Region. These initiatives encompass a range of activities, including education and school development, agriculture and livestock programs, beekeeping, housing construction, support for small businesses and microcredit schemes, infrastructure development such as water pipes and aqueducts, and fostering a local Health Care Center.

Among the promoted health care projects, special attention was given to screen for high BP. The BP screening was conducted by a local team, who received a two-day training from a certified European Hypertension Specialist (FM). The team was composed of 20 community health care workers, five community health care supervisors, and one nurse with a Bachelor Degree in General Nursing and a specific education in hypertension surveillance.

The project was approved by the Rwanda National Ethics Committee (RNEC) as “Study for better Blood Pressure and Cardiovascular Risk Control in a rural area of the District of Nyaruguru” (RNEC Approval Nr 752/RNEC/2019) and was performed in accordance with the Declaration of Helsinki. The project was endorsed by the World Hypertension League. Verbal informed consent in Kinyarwanda, the national spoken language, was obtained from all participants.

### Study design

This community-based, house-to-house BP screening was carried out between February and July 2020 in 12 villages of Mata Sector (Cyafurwe, Mata, Matyazo, Murambi, Nyamyumba, Ramba, Rimbanya, Runono, Rwamiko, Rwinanaka, Taba, and Tubururu). The remaining four villages (Gasasa, Mataba, Nyacyondo, and Ruhunga) were not involved in the screening because of important geographic distance barriers.

Data collected included demographics, self-reported previous diagnosis and/or treatment of hypertension and/or diabetes, and social history of tobacco smoking. Measurements included anthropometrics, BP, and resting heart rate.

Weight was taken using a digital Pearl^®^ platform scale (PEARL GmbH, Buggingen, Germany) and height using a 300 cm Metrica^®^ measuring tape (Metrica SpA, Milan, Italy). Body mass index (BMI), calculated as ratio between weight in kilogram and height in square meters, was used as a simple surrogate of normal (≤24.9 kg/m^2^) or excessive (≥25.0 kg/m^2^) body fat content.

According to European Society of Hypertension (ESH) Guidelines [5, 6], after resting in seated position for at least five minutes, sitting brachial BP and heart rate were measured three times one minute apart, using a validated, automated, oscillometric OMRON M6 IT-HEM-7322-E BP monitor with Intelli Wrap Cuff (HEM-FL31-E) technology (Omron Healthcare UK Ltd, Milton Keynes, UK) [7]. This pre-formed, expanded coverage BP cuff inflates around the upper arm to fit arms with a circumference of 22–42 cm and ensures accurate readings [8]. The mean of the second and third value was calculated and retained. Pulse pressure was calculated by subtracting diastolic from systolic BP.

According to the ESH Guidelines [5], BP is classified into four categories: normal (<140/90 mm Hg); grade 1 (140–159/90–99 mm Hg), grade 2 (160–179/100–109 mm Hg), and grade 3 (≥180/≥ 110 mm Hg) hypertension. We refer to this categorization of BP [5] hereafter. Fast heart rate was defined as resting heart rate >80 beats/min.

### Statistical analysis

Dichotomous categorical data are expressed as count and were analyzed by means of the *χ*^2^ test [9]. Ordered categorical data were analyzed by means of the non-parametric Kruskal-Wallis test [10]. The normality D’Agostino-Pearson Omnibus Test [11] disclosed that age (*P* < 0.0001), weight (*P* < 0.0001), height (*P* < 0.0001), BMI (*P* < 0.0001), resting heart rate (*P* < 0.0001) and both systolic and diastolic BP (*P* < 0.0001) do not follow a Gaussian distribution. Consequently, continuous data are presented as median and interquartile range and were analyzed using the non-parametric Mann-Whitney-Wilcoxon U test and the Kruskal-Wallis test [9]. Age, resting heart rate, and BMI are also presented [12] in box-and-whisker plots (the bottom and top of the box represent the 25th and 75th centile, the middle band the median, and the ends of the whiskers the 10^th^ and the 90^th^ centile). Simple regressions with the non-parametric coefficient of correlation r_s_ were also calculated [13]. Two-sided *P* values of <0.05 were considered significant. GraphPad Prism for Macintosh 10.0.2 (GraphPad Software, San Diego, California, United States) was used for statistics.

## Results

**Fig. 1:**
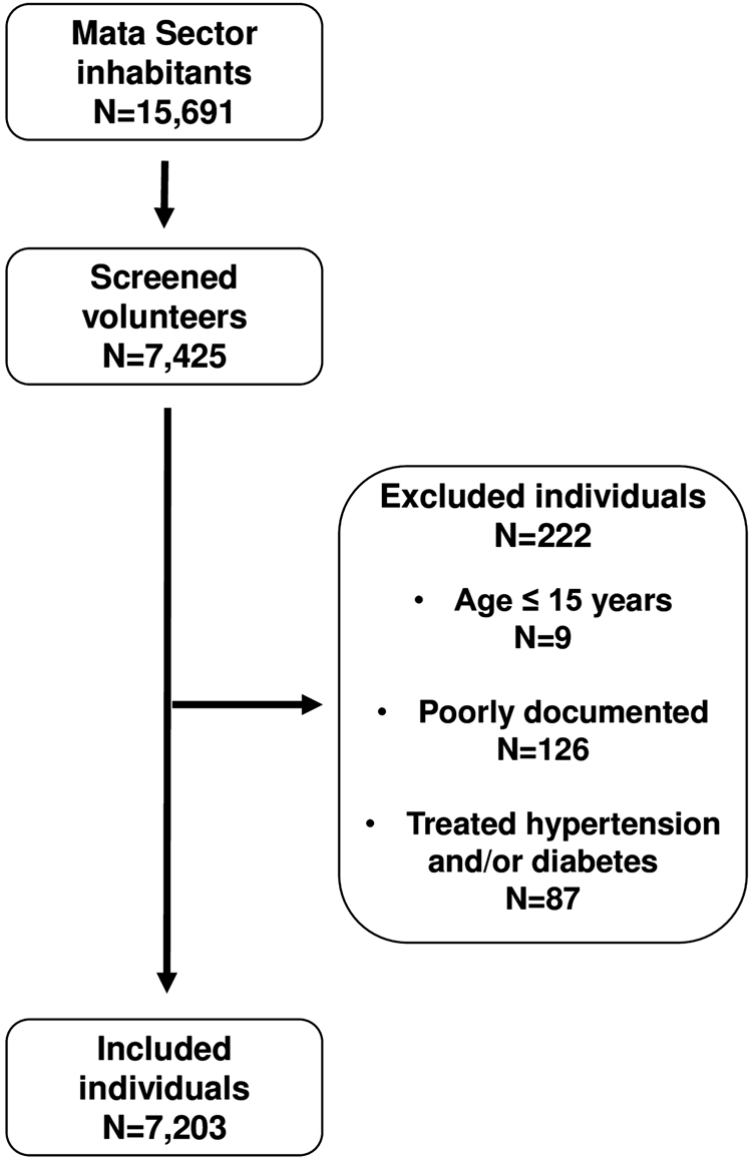
Flowchart of screening participants. Only individuals ≥15 years of age were considered for the current analysis.

The number of inhabitants who voluntarily underwent the screening was 7425. Two hundred and twenty-two individuals were excluded because of being <15 years old, missing data, being under treatment for hypertension, and reporting a diagnosis or treatment of diabetes, as depicted in Fig. 1.

**Fig. 2:**
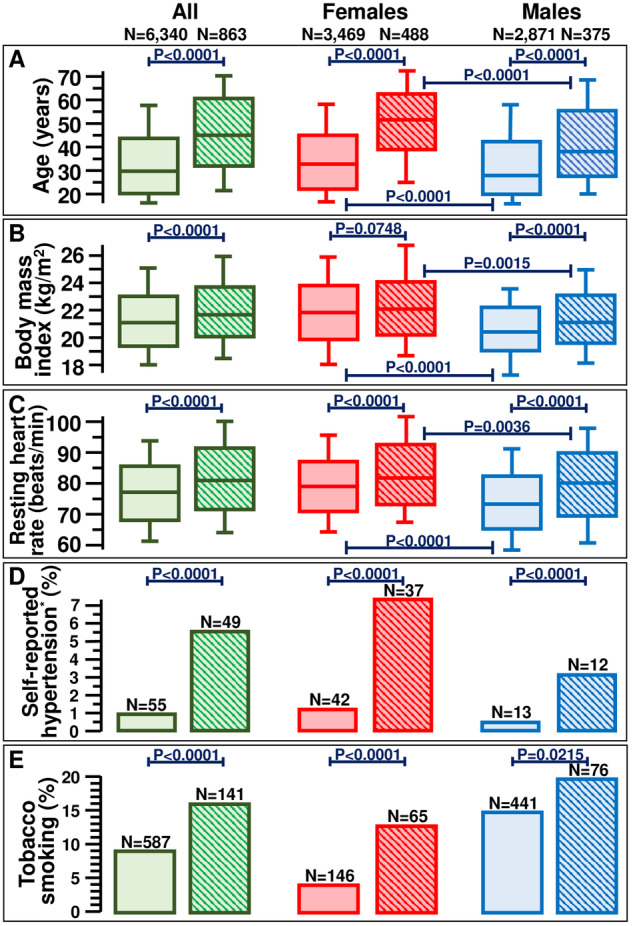
Characteristics of all 7203 participants, of which 3957 female and 3246 male individuals, undergoing the blood pressure screening in Mata Sector of Nyaruguru District (Rwanda). Age (**A**), Body mass index (**B**), Resting heart rate (**C**), Self-reported hypertension (**D**), and Tobacco smoking (**E**) in individuals with normal(   □  ) or elevated () blood pressure. Data contained in panels **A**–**C** are presented as box-and-whisker plots. *untreated.

The characteristics of the remaining 7203 participants (3957 females and 3246 males, aged 32 [21–46]) are presented in Table 1. History of untreated hypertension and tobacco smoking was positive in 1.4 and 10% of the screened population, respectively. Eleven percent had a BMI ≥ 25.0 kg/m^2^. BP was normal in 6340 (88%) and elevated in 863 (12%) participants. Grade 1 hypertensive blood pressure readings were detected in 697 (81%), grade 2 in 134 (16%), and grade 3 in 32 (3.7%) individuals. Compared to normotensive participants, individuals with hypertensive values were significantly older (46 versus 30 years; *P* < 0.0001) and more frequently reported a prior diagnosis of untreated hypertension (5.7% versus 0.9%; *P* < 0.0001) and tobacco smoking habit (16% versus 9.3%; *P* < 0.0001). Additionally, a slightly greater proportion of participants with high BP readings (14% versus 11%; *P* = 0.0019) had a BMI ≥ 25.0 kg/m^2^ (Fig. 2, panel B). A significant (*P* < 0.0001) linear correlation was found between BMI, taken as independent value, and both systolic (*y* = 0.4065 x + 110.8; rs = 0.09596) and diastolic (*y* = 0.4433 x + 65.5; rs = 0.1209) blood pressure in the whole group of 7203 participants. Lastly, also pulse pressure and resting heart rate was higher in the group of individuals with elevated BP values (*P* < 0.0001). Of note, among those found to have high BP readings, 338 (39%) had isolated systolic (systolic ≥140 mm Hg and diastolic <90 mm Hg) BP values, while 244 (28%) isolated diastolic (systolic <40 mm Hg and diastolic ≥90 mm Hg), and 281 (33%) systo-diastolic (systolic ≥140 mm Hg and diastolic ≥90 mm Hg). Pulse pressure was higher (*P* < 0.0001) in individuals with isolated systolic (66 [61–74] mm Hg) than in those with isolated diastolic (36 [29–41] mm Hg) and systo-diastolic (56 [50–66] mm Hg) blood pressure elevation.

**Table 1:** Characteristics of 7203 individuals undergoing the blood pressure screening in Mata Sector of Nyaruguru District (Rwanda).

	All	Blood Pressure		*P* value
		Normal	High	
*N* (%)	7203	6340 (88)	863 (12)	
Females : Males, *N* (%)	3957 (55): 3246 (45)	3469 (55): 2871 (45)	488 (57): 375 (43)	0.2358
Age, years	32 [21–46]	30 [20–44]	46 [32–61]	<0.0001
Self-reported hypertension^a^, *N* (%)	104 (1.4)	55 (0.9)	49 (5.7)	<0.0001
Tobacco smoking, *N* (%)	728 (10)	587 (9.3)	141 (16)	<0.0001
Weight, kg	56 [50–62]	56 [50–62]	58 [51–64]	<0.0001
Height, m	1.62 [1.56–1.68]	1.62 [1.56–1.68]	1.62 [1.56–1.68]	0.8924
Body mass index, kg/m^2^				
value	21.2 [19.5–23.1]	21.2 [19.5–23.1]	21.7 [20.0–23.7]	<0.0001
≥25.0, *N* (%)	815 (11)	688 (11)	129 (14)	0.0019
Blood pressure, mm Hg				
systolic	118 [110–127]	116 [108–124]	144 [137–153]	
diastolic	75 [68–81]	73 [68–79]	91 [84–95]	
pulse pressure	44 [37–51]	43 [37–50]	56 [42–67]	
Resting heart rate, beats/min	78 [69–87]	77 [69–86]	82 [72–92]	<0.0001

^a^untreated.

Participants with and without elevated blood pressure readings were not compared with respect to blood pressure, being statistically significant difference predictable. Data are presented as frequency (with percentage) or as median (with interquartile range).

The prevalence of individuals with high BP readings was identical in females and males (12%), as shown in Table 2. Interestingly, while in both sexes, age, pulse pressure and resting heart rate were significantly higher (*P* < 0.0001) among individuals with hypertensive than normotensive readings, BMI was significantly higher in males with hypertensive than normotensive values, but it was not significantly different (*P* = 0.0748) between females with hypertensive and normotensive values (Table 2 and Fig. 2). Age (Fig. 2, panel A), BMI (Fig. 2, panel B) and resting heart rate (Fig. 2, panel C) were higher in normotensive females than in normotensive males (*P* < 0.0001). A similar difference was observed also between females and males with elevated BP values (age: *P* < 0.0001; BMI: *P* = 0.0015; resting heart rate: *P* = 0.0036). Furthermore, being aware of their potential hypertensive status and being tobacco smokers were more common in those with high BP measurements, both in females and males (Table 2 and Fig. 2, panels D and E).

**Table 2:** Characteristics of 3957 female and 3246 male individuals undergoing the blood pressure screening in Mata Sector of Nyaruguru District (Rwanda).

	All	Blood Pressure		*P* value
		Normal	High	
**Females**				
*N* (%)	3957	3469 (88)	488 (12)	
Age, years	34 [22–48]	32 [21–45]	52 [39–63]	<0.0001
Self-reported hypertension^a^, *N* (%)	79 (2.0)	42 (1.2)	37 (7.6)	<0.0001
Tobacco smoking, *N* (%)	211 (5.3)	146 (4.2)	65 (13)	<0.0001
Weight, kg	56 [50–62]	56 [50–62]	56 [49–62]	0.4163
Height, m	1.59 [1.55–1.64]	1.60 [1.55–1.64]	1.59 [1.54–1.63]	0.2980
Body mass index, kg/m^2^				
value	21.8 [20.0–23.8]	21.8 [19.9–23.8]	21.9 [20.2–24.1]	0.0748
≥25.0, *N* (%)	629 (16)	542 (16)	87 (18)	0.3288
Blood pressure, mm Hg				
Systolic	116 [108–126]	114 [107–122]	145 [136–155]	
Diastolic	75 [69–81]	74 [69–79]	91 [86–95]	
Pulse pressure	41 [36–49]	41 [35–47]	55 [41–68]	<0.0001
Resting heart rate, beats/min	80 [72–88]	80 [72–88]	82 [74–93]	<0.0001
**Males**				
*N* (%)	3246	2871 (88)	375 (12)	
Age, years	30 [20–43]	28 [20–42]	37 [28–55]	<0.0001
Self-reported hypertension^a^, *N* (%)	25 (0.8)	13 (0.5)	12 (3.2)	<0.0001
Tobacco smoking, *N* (%)	517 (16)	441 (15)	76 (20)	0.0215
Weight, kg	57 [51–62]	57 [51–62]	60 [54–66]	<0.0001
Height, m	1.66 [1.60–1.71]	1.66 [1.60–1.71]	1.67 [1.61–1.72]	0.1052
Body mass index, kg/m^2^				
value	20.6 [19.0–22.2]	20.5 [18.9–22.1]	21.4 [19.8–23.2]	<0.0001
≥25.0, *N* (%)	186 (5.7)	146 (5.1)	40 (11)	<0.0001
Blood pressure, mm Hg				
systolic	120 [112–129]	118 [111–125]	143 [139–150]	
diastolic	74 [67–80]	72 [67–78]	90 [82–94]	
pulse pressure	46 [39–54]	46 [39–52]	58 [43–67]	<0.0001
Resting heart rate, beats/min	75 [66–84]	74 [66–83]	81 [70–90]	<0.0001

^a^untreated.

Participants with and without elevated blood pressure readings were not compared with respect to blood pressure, being statistically significant difference predictable. Data are presented as frequency (with percentage) or as median and interquartile range.

The characteristics of the 7203 screening participants according to age groups are presented in Table 3. Weight and BMI were on average significantly higher in participants with 35–44 years of age as compared to the remaining age groups. Resting heart rate and the prevalence of resting heart rate >80 beats/min were age independent. On the contrary, systolic, diastolic and pulse pressure and the prevalence of hypertensive readings were significantly age-dependent. Similarly, hypertensive grade 2 and grade 3 values were uncommon (<10%) in participants ≤34 years of age but more common (>10%) in individuals ≥35 years of age (*P* < 0.0001).

**Table 3:** Characteristics of 7203 individuals undergoing the blood pressure screening according to age groups. Data are presented as frequency (with percentage) or as median (with interquartile range).

	Age (years)						*P* values
	15–24	25–34	35–44	45–54	55–64	>65	
*N*	2465	1467	1294	833	663	481	
Females: Males, *N*	1199: 1266	792: 675	762: 532	516: 317	400: 263	288: 193	
Weight, kg	55 [49–60]	58 [53–63]	59 [53–65]	57 [51–63]	55 [50–60]	51 [46–58]	<0.0001
Height, m	1.61 [1.55–1.67]	1.62 [1.57–1.69]	1.62 [1.58–1.68]	1.62 [1.57–1.68]	1.62 [1.57–1.68]	1.60 [1.54–1.65]	<0.0001
Body mass index, kg/m^2^							
Value	20.8 [19.1–22.6]	21.7 [20.2–23.4]	22.0 [20.3–24.0]	21.4 [19.6–23.2]	20.7 [18.9–22.7]	20.2 [18.4–22.1]	<0.0001
≥25.0, *N* (%)	172 (7.0)	198 (13)	231 (18)	108 (13)	67 (10)	39 (8.1)	<0.0001
Resting heart rate, beats/min							
Value	78 [69–87]	77 [68–86]	78 [70–87]	78 [70–86]	78 [69–86]	78 [70–86]	0.0575
>80, *N* (%)	1046 (42)	570 (39)	562 (43)	337 (40)	280 (42)	188 (39)	0.1594
Blood pressure, mm Hg							
Systolic	115 [107–122]	117 [109–126]	119 [110–128]	121 [113–131]	124 [113–136]	129 [118–146]	<0.0001
Diastolic	71 [65–77]	74 [68–80]	77 [71–83]	78 [73–84]	78 [72–84]	77 [71–84]	<0.0001
Pulse pressure	44 [37–51]	43 [37–50]	42 [36–49]	43 [37–49]	45 [39–54]	52 [44–63]	<0.0001
High blood pressure, *N* (%)	112 (4.5)	131 (9.0)	162 (13)	137 (16)	153 (23)	168 (35)	<0.0001
Hypertension grade^a^, *N*							<0.0001
Grade 1	105	119	139	111	115	108	
Grade 2	7	10	21	23	30	43	
Grade 3	0	2	2	3	8	17	
Hypertension type^a^, *N*							
Isolated systolic	55	43	46	32	71	91	<0.0001
Isolated diastolic	41	51	75	39	28	10	<0.0001
Systo-diastolic	16	37	41	66	54	67	<0.0001

^a^classification of the European Society of Hypertension [5].

## Discussion

This preliminary community-based BP screening was performed among more than 7000 individuals, i.e. approximately 50% of the inhabitants of Mata Sector, a rural area in Southern Province of Rwanda. Unfortunately, four villages of Mata Sector were not screened. These remote areas were inaccessible because of long walking distances (>4 h) and limited available resources, both in terms of human capacity and financial support. The inability to reach these four villages is unlikely to have introduced a selection bias, given that people living in these areas share the same ethnicity and life conditions of those living in the other nearby villages. Our field study provides evidence that in such population hypertensive readings prevalence was 12% (without relevant differences between sexes) with a strong age dependence. Moreover, 95% of the individuals with elevated BP values were unaware of their potential hypertensive status.

**Fig. 3:**
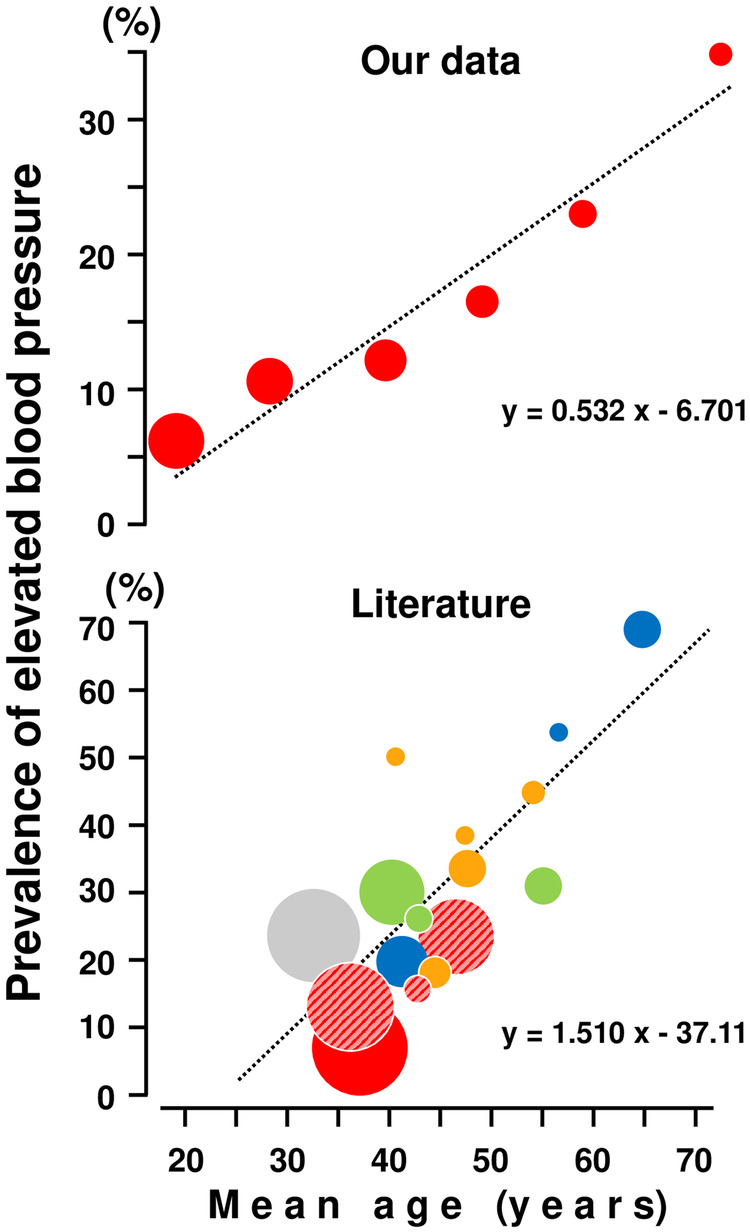
The relationship between mean age (years) and prevalence of elevate blood pressure (%). Age dependence of prevalence of elevated blood pressure in participants in this screening () and in the literature: , Democratic Republic of the Congo (*N* = 255, [15]; *N* = 730, [22]; *N* = 1000, [21]; *N* = 211, [24]; *N* = 382, [25]); , Republic of Kenya (*N* = 2111, [17]; *N* = 239, [18]; *N* = 1100, [20]); , Republic of Rwanda (*N* = 535, [14]; *N* = 5575, [19]; *N* = 4284, [26]); , United Republic of Tanzania (*N* = 928, [13]; *N* = 542, [14]; *N* = 3000, [23]), and , Republic of Uganda (*N* = 6678, [16]). The dimension of the dots is commensurate to the number of participants examined within each screening.

Between 2000 and 2022, 14 articles, encompassing 15 community-based BP screenings performed in rural East sub-Saharan Africa, were published [14–27]. The size of the screened population varied from 211 to 6678 and the mean age between 35 and 64 years. The overall prevalence of high BP ranged between 15 and 70% and was therefore higher than in our population (12%), which was on average rather young (mean age 36 years). Taking together the published studies and our data, a tendency towards an age-dependent increase in the prevalence of elevated BP appears supported by sufficient evidence (Fig. 3, lower panel). A relationship was observed also between age (when considering the six different age groups included in our study) and the prevalence of increased BP (Table 3 and Fig. 3, upper panel). Taken together, this analysis supports the notion that the increase in arterial BP is similar in Mata Sector and in the aforementioned studies carried out in East sub-Saharan Africa. Our hypothesis that the finding of low prevalence rates of elevated blood pressure in our study was associated with younger age and lower BMI, is supported by data provided by previous papers on the prevalence of hypertension in Rwanda. In fact, the overall prevalence of hypertension in Rwanda in 2018 was 15.4%; being 16.5% among males and 14.4% among females. The prevalence of hypertension was more than double in those aged 55–64 years as compared to those 44 years and below (38.6%) [20]. In another paper focusing on the prevalence of hypertension in other rural areas, rates were slightly higher (18–22%) than in our cohort, but individuals at higher age and BMI were considered [27]. Finally, hypertension prevalence was much higher in urban areas (26–34%), especially when associated with higher BMI [28].

In our population, we also observed a correlation between PP and isolated systolic blood pressure elevation. Similarly, we speculate that this observation represents the peripheral pulse amplification typical of younger individuals which was the major rate in our population [29]. It has already been stated that stroke volume is probably the major driver of the elevated PP in young individuals, whilst other hemodynamic mechanisms (presumably aortic stiffening and early return of wave reflection) are responsible for PP elevation in older individuals [30]. However, one study highlighted that also young individuals with normal stroke volume might develop sustained isolated systolic hypertension later in life because of premature aortic stiffening [31].

In high income countries, excessive body weight is strongly associated with arterial hypertension. The population included in our study was rather lean, as indicated by the fact that a BMI ≥ 25.0 kg/m^2^ was present in 11% of the participants. Nonetheless, in individuals with high BP values, the BMI and the prevalence of BMI ≥ 25.0 kg/m^2^ were slightly but significantly higher. Similar observations were reported by other studies in hypertensive populations in sub-Saharan Africa [32, 33].

Tobacco smoking significantly contributes to cardiovascular morbidity and mortality [5, 34]. The vast majority (90%) of the individuals who participated in our study were non smokers. Nevertheless, tobacco smoking was more prevalent among participants with high BP values than those with normal BP, confirming that tobacco smoking is a crucial cardiovascular modulator also in African people [35].

Fast resting heart rate predisposes to an increased cardiovascular morbidity and mortality, as recently reviewed [36]. In our study, individuals with high BP readings had a higher resting heart rate (by 5 beats/min) than the normotensive counterpart. Resting heart rate was not assessed in the previously mentioned screening studies conducted in East sub-Saharan Africa except for one of them. However, the latter study failed to report resting heart rate values in the hypertensive and in the normotensive population separately considered [15].

We have to acknowledge few limitations of our study. First, the results of our population screening, which considered BP values obtained in one visit only, are not sufficient for the diagnosis of arterial hypertension. Indeed, individuals identified with occasionally elevated BP values need further confirmatory measurements over repeated visits to establish the diagnosis of arterial hypertension [5]. Second, sedentary lifestyle, excessive alcohol consumption, adding extra salt to food, and tobacco smoking patterns, further major modulators of BP levels, were not evaluated in our study [5, 34, 37, 38]. Moreover, we were not able to investigate the relation of low prevalence of hypertension to lean body mass and probably low-sodium, high-potassium diet. A 24-hour urine collection could be indicative of dietary habits, yet unfeasible in the condition of our study. We failed to find in the literature reports about cardiovascular profile and health status of Rwanda migrates to high income countries. However, we speculate that social determinants and western lifestyle and dietary habits play a major role in hypertension development alongside with genetic predisposition, as reported for migrates from other African countries [39, 40]. Lastly, participation to this screening was voluntary thus no random selection was applied.

On the other hand, our screening has various strengths. To the best of our knowledge, this is the largest community-based BP screening ever conducted in rural sub-Saharan Africa. Despite being voluntary, the massive participation in this rather small geographical area ended up being accurately representative of the local adult population. Furthermore, it was conducted in a short time frame with the involvement of local community health care workers, who had been carefully trained and were supervised throughout the data collection. Community health care workers are crucial for conducting extended screening and tailored communication strategies for enhancing awareness about health [41]. Finally, the measurement of BP was carried out strictly adhering to recently available guidelines [6], and was performed by means of a clinically validated, automated, oscillometric device. This kind of device eliminates interobserver variability, digit preference and observer’ bias, thus resulting in a reliable and accurate estimate of BP levels. Furthermore, it does not require any calibration and transducers are quite durable, while avoiding mercury toxicity. All devices used in this study were new devices, directly made available simultaneously by the OMRON company.

In conclusion, these data substantiate that high BP, which is the leading preventable cardiovascular risk factor [3, 5, 34], and the main cause of mortality worldwide [3], represents a concerning issue within sub-Saharan Africa, where perception and awareness of cardiovascular risks and related diseases is still alarmingly low [42]. While high-income countries promote guidelines and public health campaigns for identify undiagnosed hypertension, in sub-Saharan Africa the issue is rather underrated and the commitment in prevention and management of hypertension is still mild. The rapid increase in sub-Saharan population, the rising life expectancy, the ongoing epidemiologic transition and westernization of life and dietary habits, may worrisomely lead to a quick and dramatic rise in cardiovascular disease and related-morbidity. Screening and managing high BP should be in the top priority agenda of a multidisciplinary board encompassing international public health officials, non-governmental organizations, scientists, epidemiologists and clinicians. At the same time, strategies should fit the African setting, being practical and unexpensive, and maximally inclusive.

## Summary Table

### What is known about the topic

- Hypertension is a renowned killer in Africa.
- Substantial evidence suggests that westernization of lifestyle is strongly related to the development of hypertension in urban Africa.
- Data on blood pressure values and related modulators in rural sub-Saharan Africa are scarce.

### What this study adds

- In Mata Sector, a rural region in Southern Rwanda, blood pressure is elevated in 12% of the inhabitants.
- Blood pressure correlates with age, body mass index, and resting heart rate.
- A decentralized, nurse-led blood pressure surveillance is feasible in rural sub-Saharan Africa.

## Data Availability

De-identified participant data will be made available upon reasonable request. Please contact Prof. Gianfranco Parati at gianfranco.parati@unimib.it.
